# Neuroimmune Dysregulation and AI-Driven Therapeutic Strategies in Alzheimer’s Disease

**DOI:** 10.1007/s10571-025-01651-0

**Published:** 2025-12-26

**Authors:** Shampa Ghosh, Rakesh Bhaskar, Krishna Kumar Singh, Samarth Sharma, Bhuvaneshwar Yarlagadda, Jitendra Kumar Sinha, Sung Soo Han

**Affiliations:** 1 GloNeuro, Sector 107, Vishwakarma Road, Noida, 201301 Uttar Pradesh India; 2 School of Chemical Engineering, Yeungnam University, Gyeonsang, 38541 Republic of Korea; 3 Research Institute of Cell Culture, Yeungnam University, Gyeonsang, 38541 Republic of Korea; 4 Symbiosis Centre for Information Technology, Symbiosis International (Deemed University, Rajiv Gandhi InfoTech Park, Hinjawadi, Pune, 411057 Maharashtra India; 5 GL Bajaj Institute of Technology and Management, Greater, 201308 Noida India; 6 Symbiosis Institute of Health Sciences (SIHS), Symbiosis International (Deemed University), Pune, 412115 Maharastra India

**Keywords:** Alzheimer’s disease, Artificial intelligence, Astrocytes, Blood-brain barrier dysfunction, Glial-targeted therapeutics, Immunometabolism, Inflammasome signaling, Microglia, Neuroimmune interactions, Precision medicine

## Abstract

Neuroimmune interactions have arisen as central contributors to the pathogenesis and progression of Alzheimer’s disease. The exacerbation of neuronal dysfunction in Alzheimer’s disease is collectively contributed by dysregulation of astrocytic and microglial responses, invasion of peripheral immune cells, and chronic inflammation. A mechanistic understanding of molecular, cellular, and systems-level neuroimmune interactions in Alzheimer’s disease is enabling rational, testable therapeutic strategies. We have tried to extensively address the molecular regulation of glial activation, inflammasome signaling, and immune cell invasion in Alzheimer’s disease. This review highlights microglial metabolic reprogramming and lipid-metabolism dysregulation as major drivers of neuroinflammation. Existing and future therapeutic approaches to glial activation, immunometabolism, and inflammasome signaling are thoroughly reviewed, together with novel strategies involving stem cell-derived exosomes and peripheral immunity modulation. Artificial intelligence and machine learning technologies are the latest game-changing technologies in unraveling neuroimmune complexity, discovering biomarkers, simulating disease courses, and speeding up drug discovery. Integration of multi-omics information and AI-based predictive models is suggested as a critical strategy for the development of precision medicine strategies in neuroimmune therapeutics. Advancements in neuroimmune research, coupled with computational biology technological advancements, hold the promise to transform the therapeutic environment for Alzheimer’s disease. Multitargeted, integrated interventions and data-driven strategies are set to overcome current limitations and advance closer to effective, personalized therapies.

## Introduction

 Alzheimer’s disease (AD) is a common, irreversible, and progressive neurodegenerative condition clinically defined by memory loss, cognitive decline, and behavioral changes (Scheltens et al. 2021; Sethi et al. 2024). Globally, around 49 million people aged ≥ 65 years meet criteria for AD (Xiaopeng et al. 2025). The number of people living with dementia is projected to 152.8 million by 2050 (Nichols et al. 2022). Neuropathologically, AD is defined by the extracellular amyloid-beta (Aβ) plaques, intracellular neurofibrillary tangles (NFTs) of hyperphosphorylated tau protein, synaptic disruption, and neuronal cell death (Ghosh et al. 2020; Gyimesi et al. 2024). In spite of the vast amount of progress made until now, AD remains a challenge because of the incompleteness of its intricate aetiology and because the existing treatments are not very effective. Historically, the “amyloid cascade hypothesis” has been a popular notion in the research on AD (Hardy and Allsop 1991; Hardy and Higgins 1992). The amyloid cascade hypothesis puts forward that the accumulation of Aβ peptides starts a series cascade of pathogenic processes eventually giving rise to degeneration (Nasb et al. 2024) in neural tissue. On the other hand, there has been an increasingly large body of evidence that places emphasis on the central role the neuroimmune system plays, in such a way that neuroinflammation has been shown to be a central component of overall AD pathophysiology (Kodi et al. 2024; Ji et al. 2025).

Presently, neuroinflammation is understood as the initial protective response which eliminates harmful stimuli and initiates healing processes, in the nervous system. When the process of neuroinflammation becomes chronically engaged, it becomes pathogenic. It further gets into exacerbating neuronal damage and advancing disease pathogenesis (Bhusal et al. 2022). The neuroimmune system is characterized by the dynamic interaction between the neurons and other non-neuronal cells, like microglia and astrocytes. Microglia, which are resident immune cells within the brain, have a role in the central nervous system (CNS) environment that is continuously regulated and responds quickly to any disturbances that arise (Abellanas et al. 2025). Microglia also have a dramatic change in both the form as well as their function once activated by pathological conditions like AD. These activated microglia have been demonstrated to adopt a range of phenotypes. They were previously typified as M1 (pro-inflammatory/neurotoxic) and M2 (anti-inflammatory/neuroprotective) phenotypes (Gao et al. 2023). More recent evidence indicates that there are several distinct microglial activation phenotypes (context-dependent microglial states) (Escartin et al. 2021), including disease-associated microglia (DAM) (Bhusal et al. 2022). Astrocytes, the most abundantly represented glial cells, are tasked with a range of activities that are of the highest importance. These range of tasks include the provision of support at the synaptic level, the upkeep of the blood-brain barrier (BBB), and the supply of metabolic supplements to neurons (Schiera et al. 2024). Astrocytes experience a transformation called astrogliosis that is marked by a shift both in their form and in their functional status, with neurodegenerative ailments. Reactive astrocytes, similar to microglia, were previously described as of two phenotypes, namely A1 (neurotoxic) and A2 (neuroprotective) (Lawrence et al. 2023) but as per latest nomenclature, reactive astrocyte states terminology should be used (Sofroniew 2020; Baskakov 2021; Paolicelli et al. 2022). Yet, based on recent evidence, it appears that there have been several intermediately and mixture activation states through which reactive astrocytes could go (Heneka et al. 2024; Mohammad et al. 2024). According to Wu and Eisel (2023), astrocytes share a close interaction with microglia, thus allowing them to have a great influence on the inflammatory environment as well as on the status of neuronal well-being through complex cell crosstalk mechanisms (Wu and Eisel 2023). Crosstalk between microglia and astrocytes plays an essential role in modulating neuroimmune responses in AD (Yang et al. 2020). This involves the interaction of the engagement of vast numbers of molecular mediators such as cytokines (such as IL-1β, IL-6, and TNF-α), chemokines, and complement proteins (Castellani and Schwartz 2020; Bhusal et al. 2022). To exemplify, microglial-produced cytokines and chemokines can modulate reactive astrocyte phenotypes, thus impacting neuronal survival and functioning (Bhusal et al. 2022; Mohammad et al. 2024).

Furthermore, recent evidence suggests that the activation of inflammasomes, specifically microglial NLRP3 inflammasome, is the cause of neuroinflammation associated with AD and also affects the activation of astrocytic cells and neuron survival (Wu and Eisel 2023; Kodi et al. 2024). Cognitive performance, synaptic integrity, and neuronal function are all significantly affected when there is neuroimmune dysregulation of glial cells at both cellular and molecular levels (Tastan and Heneka 2024). In addition, the therapeutic relevance of these interactions is increasingly becoming an important consideration. Indeed, identification and targeting of specific neuroimmune pathways and chemicals that are crucial to the dialogue between microglia and astrocytes hold the potential to bring novel treatment options to Alzheimer’s disease (Zhang et al. 2024).

Recent advances in artificial intelligence and machine learning (AI/ML) are enabling integration of longitudinal clinical data and multi-omics to forecast disease trajectories and stratify patients. Outside neurology, prospective-grade exemplars now predict risks across > 1,000 diseases from routine health records (Delphi-2 M), provide actionable survival curves from a single ECG in international cohorts (AIRE), deliver multimodal survival prediction in oncology (Owkin), and translate trajectory models into clinician-facing decision support (TrajVis)(Schutte et al. 2022; Li et al. 2024; Sau et al. 2024; Shmatko et al. 2025). Recent brain single-cell and spatial multi-omics resources, coupled with interpretable machine learning, enable cross-modal integration (genome = > transcriptome = > proteome = > lipidome) to define glial-immune endotypes, nominate targets, and support trial stratification(Johnson et al. 2022; Gabitto et al. 2024). The computational tools allow us to conduct integrated multi-omics and high-throughput screenings, which can potentially reveal novel biomarkers, drug targets, and pathways that play vital roles in the pathophysiology and treatment of AD (Lin et al. 2024). Plasma/CSF p-tau (e.g., p-tau217), GFAP, NfL, sTREM2, and YKL-40 provide diagnostic/prognostic information and index astroglial/microglial activity. Anchoring these to glial state signatures and inflammasome/complement activity refines patient stratification and outcome measures. When used for the research in neuroimmunology, the introduction of AI and ML methods would help us in greatly improving our understanding of AD, as well as the treatment thereof. This article gives a thorough overview of the current information on neuroimmune interactions in AD with specific attention to the roles that microglia and astrocytes have to play. We also mention the emerging treatment strategies aimed at these interactions and point out the promise of integrating AI and ML approaches to advance AD research. This would also be helpful as a long-term aim in the creation of effective neuroimmune-targeted therapeutics.

## Molecular Mechanisms of Neuroimmune Dysregulation in Alzheimer’s Disease

**Fig. 1:**
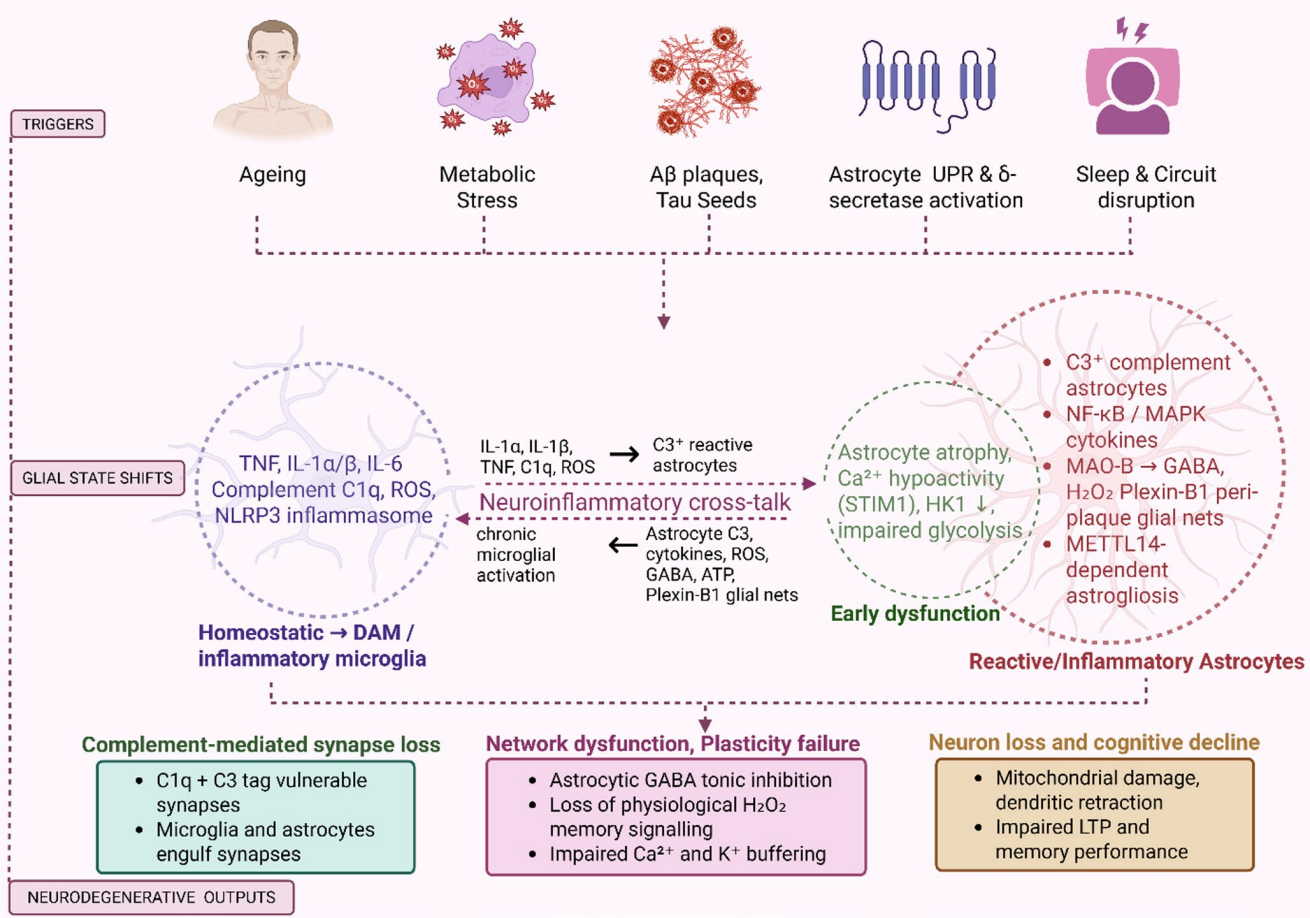
Central roles of microglia and astrocytes in neuroinflammation and neurodegeneration. Ageing, metabolic stress, amyloid-β (Aβ) plaques, tau seeds, astrocyte unfolded protein response with δ-secretase activation, and sleep or circuit disruption converge to activate microglia and astrocytes. Microglia shift from homeostatic to disease-associated states, releasing TNF, IL-1α/β, IL-6, complement C1q, reactive oxygen species and NLRP3 inflammasome products. These signals convert astrocytes from an early dysfunctional state, characterised by atrophy, Ca²⁺ hypoactivity linked to STIM1 and HK1-related metabolic impairment, into C3⁺ reactive astrocytes with NF-κB/MAPK-dependent cytokine production, MAO-B-derived GABA and H₂O₂, Plexin-B1 peri-plaque glial nets and METTL14-dependent astrogliosis. Astrocyte-derived C3, cytokines, ROS, GABA, ATP and extracellular matrix cues then sustain chronic microglial activation, forming a bidirectional neuroinflammatory loop. Both glial cell types cooperate in complement-mediated synapse pruning through C1q and C3 tagging and engulfment of vulnerable synapses. Together with astrocyte-driven disruption of physiological redox signalling and Ca²⁺ and K⁺ buffering, this loop results in network dysfunction, impaired long-term potentiation and progressive neuronal loss that underlies cognitive decline. Created in BioRender. Singh, D. (2025) https://BioRender.com/dg19m22

Microglial activation in AD entails multifaceted transitions between context-dependent states, including the newly described “disease-associated microglia” (DAM; see Box 1), which appear in reaction to amyloid pathology (Deczkowska et al. 2018; Martins-Ferreira et al. 2025). DAM display downregulation of homeostatic genes (e.g., CX3CR1, TMEM119) and upregulation of phagocytic and lipid metabolism genes like APOE, TREM2, and LPL (Gao et al. 2019; Labuda et al. 2024). This phenotype is expressed within a two-stage activation model, where TREM2 signaling is crucial for transitioning from stage 1 to stage 2 DAM, and this enables successful encapsulation and clearance (Keren-Shaul et al. 2017; Deczkowska et al. 2018). Interruption of these signalling cascades, particularly due to TREM2 mutations or the presence of the APOE4 gene, impedes this transition, leading to inadequate Aβ clearance and persistent inflammation. Parallel to this, inflammasome activation, specifically NLRP3, evokes microglial pyroptosis and ongoing cytokine release, further destabilizing neuron–glia homeostasis. Cumulatively, these changes remodel microglia from surveillant sentinels to forces advancing pathology in Alzheimer’s disease. Figure 1 summarises how ageing and proteinopathy driven triggers engage bidirectional microglia–astrocyte neuroinflammatory loop that ultimately converges on complement mediated synapse loss and neuronal degeneration.

### Microglial Activation

Microglia, the CNS-resident immune cells, play key functions in the maintenance of neuronal homeostasis. Microglia under normal states examine the environment of the neurons, with actions of synaptic pruning, apoptotic cell clearance, and the secretion of neurotrophic factors (Mohammad et al. 2024). Microglial homeostasis is also maintained by a delicate equilibrium between context-dependent microglial states. Nevertheless, under pathological conditions like AD, microglia adopt more pro-inflammatory phenotypes, profoundly changing their functions and roles (Table 1). Due to the persistent assault of Alzheimer’s-related danger signals on microglia, these cells undergo a molecular “gear-shift”: they deactivate the homeostatic surveillance gene program responsible for maintaining cerebral order and activate stress-, complement-, and cytokine-dominated programs instead. This induces the highly inflammatory disease-associated microglia or microglial neurodegenerative phenotype (DAM/MGnD) state, which excels at producing pro-inflammatory signals (TNF-α, IL-1β, C1q, ROS) but increasingly falters in the essential functions of synapse remodelling and amyloid clearance (Xu et al. 2022). Microglial phenotypes vary from homeostatic/surveilling, activated (pro-inflammatory), and DAM states (Nimmerjahn et al. 2005; Mavroudis et al. 2020). Homeostatic microglia are responsible for brain health with ongoing surveillance. In response to pathological stimuli, the cells are activated, migrating into pro-inflammatory states with amplified secretion of cytokines like TNF-α, IL-1β, and IL-6, that may worsen neuronal damage (Bhusal et al. 2022). DAM, newly recognized in neurodegenerative settings, have a unique gene expression profile with upregulation of certain genes such as TREM2, APOE, CST7, and CD68, and downregulation of homeostatic genes such as CX3CR1 and P2RY12 (Keren-Shaul et al. 2017). Damaged neurons are potent microglial activators. Focal neuronal injury and network hyperexcitability release extracellular ATP that rapidly recruits and reorients microglial processes via P2Y12 signaling, producing process convergence at stressed axons and synapses. In Alzheimer’s-relevant contexts, ATP and other DAMPs cooperate with plaque-associated cues to drive local chemotaxis and state transitions, linking neuronal stress to microglial reprogramming and inflammatory amplification (Davalos et al. 2005; Eyo et al. 2014; Haynes et al. 2006).

**Table 1:** The neuroimmune triggers in alzheimer’s disease, the principal microglial and astrocytic sensors implicated, their downstream mechanisms, and representative therapeutic targets supported by primary evidence

Trigger/source	Principal microglial sensors	Key downstream mechanisms/pathways	AD-relevant primary evidence	Therapeutic targets/examples
Aggregated Aβ (oligomers/plaques)	TREM2–DAP12/SYK; TAM receptors (Axl/Mer via Gas6/Protein S); pattern-recognition: CD14/TLR2/TLR4; scavenger receptors (e.g., CD36)	Transition to disease-associated states; motility/encapsulation of plaques; NF-κB–driven cytokines; phagocytosis and plaque compaction	TREM2 risk variants increase AD risk (Guerreiro et al. 2013) and TREM2 sustains lipid sensing in Aβ models (Wang et al. 2015); TAM receptors promote plaque containment/compaction; fibrillar Aβ activates microglia via CD14/TLR2/TLR4 (Reed-Geaghan et al. 2009; Jonsson et al. 2013; Long et al. 2024)	TREM2 agonist antibody AL002 (first-in-human Phase 1 with target engagement); conceptual TLR modulators.
Tau fibrils/oligomers	NLRP3 inflammasome in microglia (priming via NF-κB, activation via tau)	ASC speck formation; IL-1β/IL-18 maturation; feed-forward glial activation; tau pathology amplification	Tau activates NLRP3; Nlrp3−/− mice show reduced tau pathology and cognitive deficits (Reed-Geaghan et al. 2009; Ising et al. 2019)	NLRP3 inhibitors: MCC950 (tool compound) and dapansutrile/OLT1177 (clinical-stage). (Coll et al. 2015; Sánchez-Fernández et al. 2019; Lonnemann et al. 2020)
Complement-opsonized synapses (neuronal C1q/C3 tagging)	CR3 (CD11b/CD18) on microglia; C1q/C3 at synapses	Activity-dependent synaptic pruning; early synapse loss around Aβ plaques	Complement mediates physiological synapse elimination (Stevens et al. 2007) microglial CR3/C1q drive early synapse loss in AD models (Hong et al. 2016)	C1q blockade (ANX005; preclinical/early clinical), complement pathway inhibitors (Lansita et al. 2017).
Damaged/stressed neurons (DAMPs): ATP/UDP; HMGB1; exposed phosphatidylserine	P2Y12 (ATP sensing) for chemotaxis; P2Y6 (UDP) for phagocytosis; TAM receptors recognize PtdSer via Gas6/ProS	Rapid process extension to injury; engulfment of stressed elements; cytokine induction	ATP released from injury drives rapid microglial process extension via P2Y12 (Davalos et al. 2005; Haynes et al. 2006); UDP–P2Y6 triggers phagocytosis (Schwander et al. 2007)	Experimental: P2Y6 antagonism (preclinical); microglial chemotaxis modulators. (Dundee and Brown 2024)
Microglia–astrocyte cytokines (IL-1α, TNF, C1q from microglia) → reactive astrocytes	Cytokine receptors on astrocytes; complement receptors	Induction of neurotoxic reactive astrocytes; loss of homeostatic astrocyte functions; neuron death	Microglia-derived IL-1α/TNF/C1q induce neurotoxic reactive astrocytes; blocking these factors prevents toxicity (Liddelow et al. 2017)	Upstream cytokine neutralization (experimental); complement interception (Liddelow et al. 2017)
Homeostatic-to-disease-associated microglial state transition (lipid/apolipoprotein cues)	TREM2 binding to APOE/CLU; lipid sensing	Metabolic reprogramming; proliferation; barrier formation around plaques	DAM program in AD models (Keren-Shaul et al. 2017); human AD microglia show distinct activation (HAM) (Srinivasan et al. 2020); TREM2 binds APOE/CLU (Zheng et al. 2018)	
Colony-stimulating factors (CSF1/IL-34)	CSF1R	Survival and proliferation of microglia	CSF1R blockade eliminates microglia in vivo (Elmore et al. 2014); microglial elimination modifies AD phenotypes (Dawe et al. 2016)	

Examples of therapeutic targets are investigational unless otherwise indicated. *Aβ* Amyloid-β, *DAM* Disease-associated microglia, *HAM* Human AD microglia, *DAMP* Damage-associated molecular pattern, *NF-κB* Nuclear factor-κB, *NLRP3* NOD-, LRR- and pyrin domain-containing protein 3, *ASC* Apoptosis-associated speck-like protein containing a CARD;* CSF1R* Colony-stimulating factor 1 receptor,* CR3* Complement receptor 3,* P2Y12/P2Y6* Purinergic receptors,* SYK* Spleen tyrosine kinase,* TAM* Tyro3/Axl/Mer receptor tyrosine kinases,* Gas6* Growth-arrest–specific 6, * ProS* Protein S,* APOE* Apolipoprotein E,* CLU* Clusterin

Box 1 | Disease-associated microglia (DAM) vs. human AD microglia (HAM) In mouse AD models, single-cell RNA-seq defined disease-associated microglia (DAM) as a two-step response in which homeostatic/surveilling genes (e.g., P2ry12, Tmem119, Cx3cr1) decline while lipid handling and phagocytic modules rise; the mature, TREM2-dependent phase prominently induces genes such as Lpl, Itgax, Cst7, Apoe and related receptors (Keren-Shaul et al. 2017). In human Alzheimer’s cortex, human AD microglia (HAM) show a profile that only partially overlaps DAM: HAM display an enhanced aging signature with APOE upregulation and other disease-related programs, while many canonical DAM genes are not induced to the same extent; the data emphasize species and context differences and caution against equating mouse DAM with human AD microglia(Srinivasan et al. 2020).

Rare heterozygous coding variants in TREM2 (for example p.R47H) confer a substantial increase in risk of late-onset Alzheimer’s disease, as shown independently by two large case–control sequencing studies in 2013 (Guerreiro et al. 2013; Jonsson et al. 2013). In contrast, the common APOE ε4 allele shows a strong gene-dose effect on AD risk in late-onset families (Corder et al. 1993). Mechanistic studies demonstrate that TREM2 is required for effective microglial responses to Aβ and damage-associated lipids: TREM2 deficiency or risk-variant function impairs lipid sensing, microglial clustering at plaques, chemotaxis, and metabolic fitness, thereby increasing amyloid seeding and exacerbating neuritic pathology in vivo (Jay et al. 2015; Wang et al. 2015; Mazaheri et al. 2017; Ulland et al. 2017; Parhizkar et al. 2019). TREM2 binds apolipoproteins (including APOE and CLU/APOJ) and drives an APOE-dependent transcriptional reprogramming of microglia from homeostatic to disease-associated states, providing a direct mechanistic axis that connects genetic risk to microglial phenotype and plaque containment (Atagi et al. 2015; Yeh et al. 2016; Krasemann et al. 2017). TREM2 enhances phagocytic removal of amyloid-β (Aβ) plaques and regulates inflammatory responses of microglia (Ulrich et al. 2014; Chen et al. 2025). Mutations or loss of TREM2 impair activated microglial function, diminishing clearance functions and enhancing AD pathology (Wu and Eisel 2023). Likewise, the APOE ε4 allele is a major genetic risk for AD, affecting microglial activation, neuroinflammation, and Aβ deposition, reinforcing the essential contribution of genetic control to microglial functions (Wolfe et al. 2018; Ferrari-Souza et al. 2023).

### NLRP3 Inflammasome

**Fig. 2:**
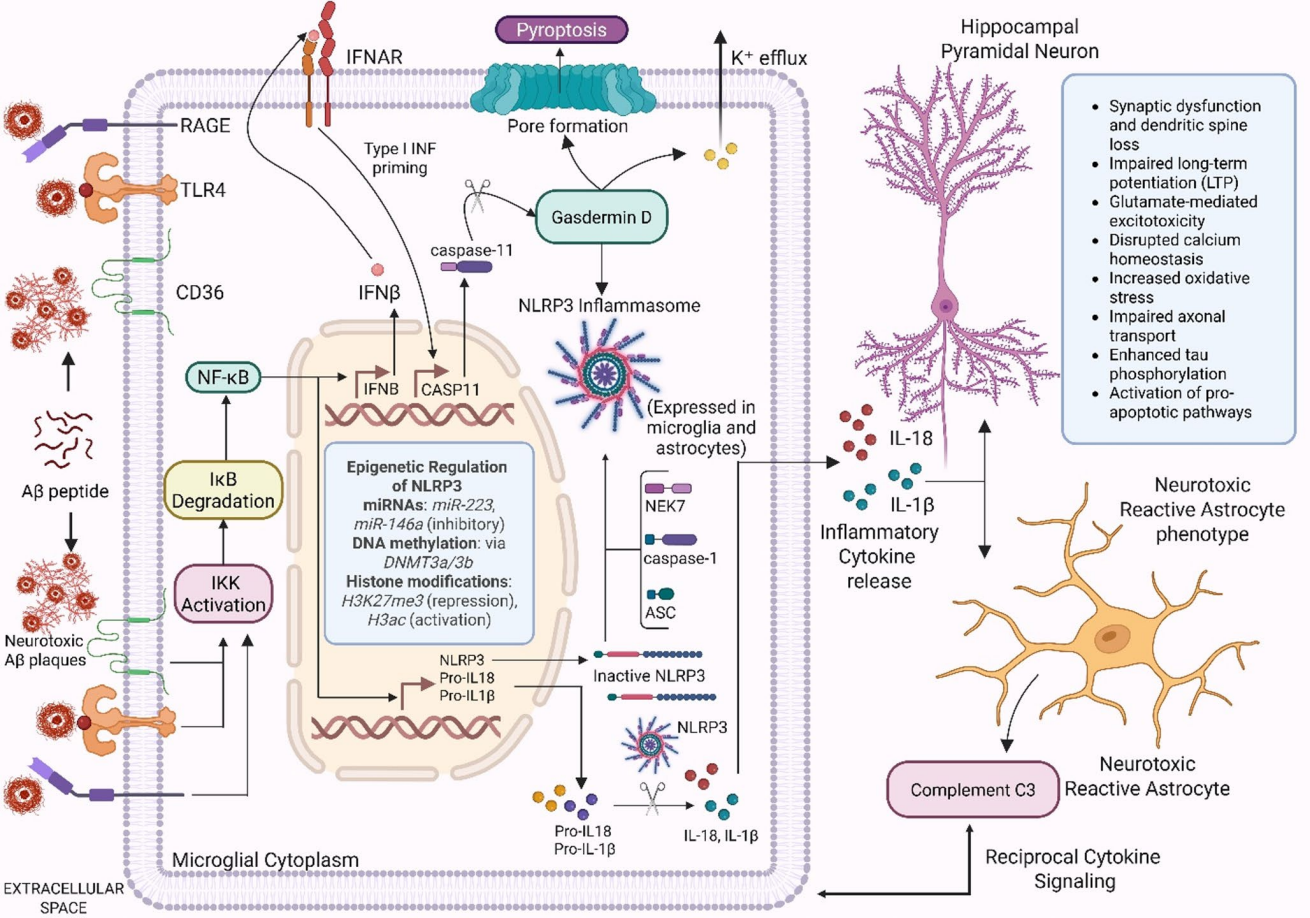
Canonical and Non-Canonical Activation and Regulation of the NLRP3 Inflammasome in Alzheimer’s Disease Aggregated amyloid-beta (Aβ) peptides interact with microglial pattern recognition receptors including TLR4, CD36, and RAGE, activating the NF-κB signaling pathway. This transcriptionally primes the microglial nucleus to express NLRP3, pro-IL-1β, and pro-IL-18 (Signal 1). Concurrent cellular stress signals such as potassium efflux, lysosomal rupture, or mitochondrial dysfunction initiate NLRP3 inflammasome assembly (Signal 2), involving NLRP3, ASC, NEK7, and caspase-1. This leads to the cleavage and release of mature IL-1β and IL-18. These cytokines contribute to synaptic dysfunction, tau pathology, and neuronal degeneration, while also inducing reactive A1 astrocytes that produce complement C3 and maintain a pro-inflammatory environment. In the non-canonical pathway, intracellular stimuli upregulate IFNβ, which primes the expression of caspase-11 through type I interferon signaling. Caspase-11 cleaves gasdermin D, forming membrane pores that cause potassium efflux, a potent trigger for canonical NLRP3 activation. Epigenetic regulation of NLRP3 includes DNA methylation via DNMT3a/3b, histone modifications such as H3K27me3 and H3ac, and microRNAs including miR-223 and miR-146a, all of which modulate microglial inflammasome activity. Together, these pathways drive chronic neuroinflammation in Alzheimer’s disease. Created in BioRender. Singh, D. (2025) https://BioRender.com/1qxnrxd

The NLRP3 (NOD-like receptor protein 3) inflammasome is a cross-cell inflammatory hub: it assembles primarily in microglia yet conditions astrocytic reactivity through IL-1 family cytokines and damage signals, thereby bridging innate immune activation across glia. The NLRP3 inflammasome is known to be a key effector of innate immunity, which is significantly involved in AD-related neuroinflammation (Terzioglu and Young-Pearse 2023). The NLRP3 inflammasome structurally consists of NLRP3 protein, apoptosis-associated speck-like protein with a caspase recruitment domain (ASC), and pro-caspase-1 (Venegas et al. 2017; Kelley et al. 2019; Ye et al. 2023). Both canonical and non-canonical activation mechanisms are followed by the inflammasome (Leung et al. 2022) (Fig. 2). The canonical pathway involves the recognition of many danger signals such as aggregated Aβ by NLRP3, leading to the recruitment of ASC, caspase-1 activation, subsequent cleavage and maturation of pro-inflammatory cytokines IL-1β and IL-18 (Heneka et al. 2018). The non-canonical pathway involves mouse caspase-11 (equivalent to human caspase-4/5), which indirectly activates the canonical inflammasome, hence augmenting inflammation (Mohammad et al. 2024). The activation of the NLRP3 inflammasome is essential for the course of the illness in AD. Activation of microglial NLRP3 triggers neuroinflammation and contributes to Aβ and tau pathology (Ising et al. 2019; Van Zeller et al. 2021).

Increased inflammasome activity is known to worsen the astrocytic activation, and contributing to an inflammatory microenvironment as well as neuronal dysfunction (Wu and Eisel 2023). Epigenetic pathways are being increasingly known to modulate inflammasome function which affects the NLRP3 expression and function in AD. DNA methylation and histone modifications can also regulate NLRP3 transcription levels, thereby modulating inflammatory reactions (Raneros et al. 2021). Additionally, recent evidence suggests that miRNAs play a crucial role in regulating NLRP3 expression as well, implying that epigenetic interventions could offer therapeutic options to manage the inflammasome-mediated neuroinflammation in AD (Heneka et al. 2018; He et al. 2025; Swanson et al. 2019). Interestingly, the expression of NLRP3 is often maintained at low levels due to the presence of conserved binding sites for many miRNAs (like miR-223, miR-7, and miR-22) inside its 3′-UTR (Table 2). When a corresponding miRNA is plentiful, it binds to the 3′-UTR, inhibits ribosomal loading, and hastens the transcript degradation, hence impeding the priming phase of the concerned inflammasome. In AD, several miRNAs are aberrantly expressed, disrupting the equilibrium and promoting persistent NLRP3 priming and the production of pyroptotic cytokines (Hu et al. 2024).

**Table 2:** Micro-RNAs that modulate the NLRP3 inflammasome in alzheimer’s disease

miRNA	Expression Change in AD	Validated Mechanism	Key Data Summary	References
miR-22-3p	↓ in serum & hippocampus of AD patients and APP/PS1 mice	Binds Sox9/NF-κB, represses NLRP3 transcription; exosomal delivery reduces GSDMD cleavage and IL-1β/IL-18 release	Downregulated in both AD brain and blood. Inverse correlation with NLRP3 and GSDMD. Mechanism validated via Sox9 targeting and NF-κB suppression. adipose-derived MSC exosomes loaded with miR-22-3p restored cognition in APP/PS1 mice.	(Han et al. 2020; (Kim et al. 2021); Zhai et al. 2021; Xia et al. 2022)
miR-212-3p	↓ in cortex and CSF of AD patients and Aβ-injected rats	Targets SP1, indirectly downregulates BACE1 and suppresses NLRP3 expression; agomir injection reduces IL-1β and improves memory	Cortical and CSF downregulation. SP1/BACE1/NLRP3 axis validated in Aβ rat models. Hippocampal injection of agomir reduced IL-1β and reversed memory impairment.	(Nong et al. 2022; Pichler et al. 2017)
miR-204/miR-373	↓ in neuron-derived plasma exosomes of AD patients	Loss of these miRNAs correlates with plaque-adjacent microglial NLRP3 expression; in vitro replacement suppresses IL-1β	Reduced in neuron-derived exosomes in AD serum. Mechanistic link to NLRP3-TXNIP axis inferred; IL-1β suppression shown in vitro, not yet validated in vivo.	(Taşdelen et al. 2022; Wu et al. 2021)
miR-223-3p	↑ in PBMC; sequestered in plaque-reactive astrocytes by lncRNA SNHG14	Targets NLRP3 3′-UTR; AVE0991 restores miR-223-3p repression by disrupting SNHG14 sponge	Elevated in PBMC of AD patients but functionally suppressed in astrocytes due to SNHG14. AVE0991 treatment restores activity, reduces NLRP3 and improves pathology in APP/PS1 mice.	(Duan et al. 2021; La Rosa et al. 2021)
miR-7-5p	↑ in AD blood; ↓ in cortex	Binds NLRP3 3′-UTR; stavudine (D4T) modulates miR-7-5p and miR-223-3p, suppressing inflammasome	Cortical downregulation and peripheral upregulation reported. Direct targeting of NLRP3 validated. D4T modulates miR-7-5p/miR-223-3p and attenuates inflammasome activation in patient-derived PBMCs.	(La Rosa et al. 2021; Pichler et al. 2017)

Highlighted are miRNAs whose expression shifts in Alzheimer’s disease patient tissue or APP/PS1 mouse brains, whose ability to suppress NLRP3 signalling is mechanistically proven, and whose reactivation—via exosomal delivery, agomirs, or targeted small molecules—has been shown to quell neuroinflammation and improve cognitive outcomes. ↓/↑ = decrease/increase versus age-matched controls; *APP/PS1* Transgenic amyloid mouse model, *PBMC* Peripheral blood mononuclear cells; *MSC* Mesenchymal stromal cell, *GSDMD* Gasdermin-D, *D4T* Stavudine, *BACE1* β-site APP-cleaving enzyme 1, *SP1* Specificity protein 1; *Sox9* SRY-box 9; *TXNIP* Thioredoxin-interacting protein

### Astrocytic Responses

**Fig. 3:**
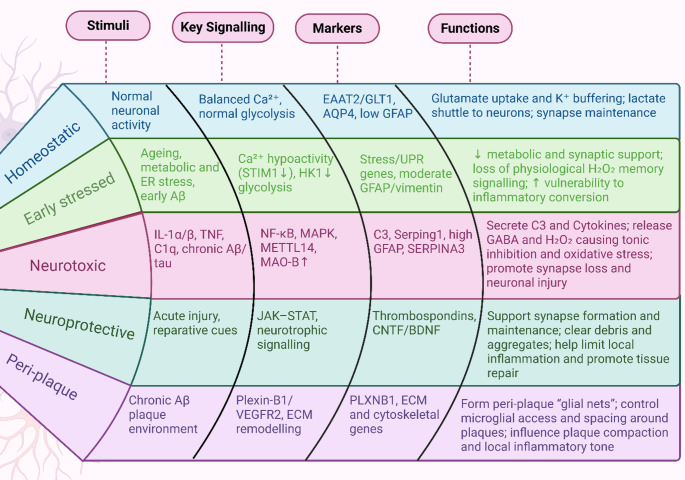
Comparison of astrocyte reactive phenotypes in neurodegeneration. Schematic fan plot summarising major astrocyte phenotypes described in neuroinflammatory and neurodegenerative conditions. Rows depict homeostatic, early stressed, neurotoxic (A1/DAA-like), neuroprotective (A2-like) and peri-plaque (PLXNB1⁺) astrocytes, while columns indicate typical stimuli, key signalling pathways, markers, and principal functions. Homeostatic astrocytes are driven by normal neuronal activity, show balanced Ca²⁺ signalling and intact glycolysis, express EAAT2/GLT1, AQP4 and low GFAP, and support glutamate and K⁺ homeostasis, lactate supply and synapse maintenance. Early stressed astrocytes arise with ageing, metabolic and ER stress or early Aβ exposure, exhibit Ca²⁺ hypoactivity and HK1-related glycolytic impairment, upregulate stress/UPR markers and provide reduced metabolic and synaptic support, increasing vulnerability to inflammatory conversion. Neurotoxic astrocytes are induced by IL-1α/β, TNF and C1q in the context of chronic Aβ and tau, activate NF-κB/MAPK, METTL14 and MAO-B, express C3, Serping1 and high GFAP/SERPINA3, and secrete C3, cytokines, GABA and H₂O₂ that drive synapse loss and neuronal injury. Neuroprotective astrocytes emerge in reparative settings, engage JAK–STAT and neurotrophic signalling, express thrombospondins and CNTF/BDNF, and support synapse formation, debris clearance and tissue repair. Peri-plaque PLXNB1⁺ astrocytes in chronic Aβ environments signal via Plexin-B1/VEGFR2 and ECM pathways, express PLXNB1 and cytoskeletal/ECM genes, form peri-plaque “glial nets” and regulate microglial access, plaque compaction, and the local inflammatory milieu. Created in BioRender. Singh, D. (2025) https://BioRender.com/uu06dqd

Astrocytes are abundant CNS glia that regulate neurotransmission, provide metabolic support, help maintain the blood–brain barrier, and shape immune signaling. Reactive astrocytes comprise diverse, context-dependent states defined by transcriptomic, morphological, and functional changes. Current consensus discourages binning into A1/A2 and recommends describing stimuli, features, and context explicitly (Escartin et al. 2021; Paolicelli et al. 2022; Santos et al. 2025). Microglia-derived cytokines, particularly IL-1α, TNF, and C1q, can induce inflammatory/reactive astrocyte programs marked by complement C3 upregulation and synapse-modulating outputs (Liddelow et al. 2017). Cytokines such as IL-1β and TNF-α further augment astrocytic reactivity and can exacerbate neuroinflammatory cascades. In parallel, metabolic coupling and lipid handling between microglia and astrocytes constrain or amplify inflammation and influence synaptic support (Mi et al. 2023). The interactions between astrocytes and microglia constitute an important aspect of neuroimmune dynamics in the pathophysiology of AD (Deng et al. 2024). Importantly, activated microglia is known to produce certain cytokines that stimulate astrocytic responses, and hence resulting into a cascade of chronic inflammation processes. Whereas, microglial interleukin-1β (IL-1β) and tumour necrosis factor-alpha (TNF-α) significantly augment astrocytic reactivity, and this adversely affect the microglial function and further intensify various inflammatory processes (Wu and Eisel 2023). As per the current understanding, cytokine and chemokine signalling significantly influence different glial connections and neuroinflammation in the aetiology of AD. Reactive astrocyte phenotypes, from early stressed to neurotoxic, neuroprotective and peri-plaque states, are compared in Fig. 3.

## Cellular and Systems-Level Neuroimmune Dynamics

Alzheimer’s disease at the cellular and systemic levels is understood by subtle interactions between CNS-resident immune cells and peripheral immune constituents. Neuroimmune interactions include complex dynamics that include immune cell infiltration across breached barriers, metabolic reprogramming of immune cells, and the persistent cross-talk between microglia, astrocytes, and peripheral immune cells that together contribute to neuroinflammation and AD development (Kölliker-Frers et al. 2021; Müller et al. 2025).

### Immune Cell Infiltration

A healthy blood-brain barrier (BBB) is crucial for its responsibility for preserving CNS homeostasis to a large level (Segarra et al. 2021). This is done by keeping the entry of peripheral immune cells and molecules tightly regulated from peripheral circulation. In AD, the integrity of BBB has been observed to be disrupted, allowing the undesirable infiltration of these peripheral immune cells into the brain parenchyma (Bhusal et al. 2022; Xingi et al. 2023). In addition to that, dysfunctional endothelium, diminished expression of tight junction proteins, and inflammatory mediators all contribute to the disruption of the BBB permeability. This breakdown of the barrier enables immune cells like CD4 + and CD8 + T lymphocytes, regulatory T cells (also known as Tregs), B cells, natural killer (NK) cells, and peripheral macrophages to invade the CNS (Castellani and Schwartz 2020; Gate et al. 2020). CD4 + T cells normally perpetuate inflammation by secreting cytokines, while CD8 + cytotoxic T cells have the potential to cause neuronal damage. CD4 + cells are not intrinsically destructive; the ratio of pro-inflammatory Th1/Th17 to counter-regulatory Th2/Treg signals determines whether they perpetuate or resolve glial activation (Xie et al. 2023). CD4 + T cells dictate the inflammatory tone through their cytokine profile, while CD8 + T cells convert that tone into direct neuronal damage when the “do-not-kill” MHC-I silence is removed (Zhang et al. 2023). Targeting chemokines, cytokines and checkpoint pathways that bias these T-cell responses is true potential to tone down inflammasome-mediated neurodegeneration without crippling systemic immunity (Dias-Carvalho et al. 2023). Tregs, however, can potentially hold back overinflammation, though they are themselves dysregulated in the context of AD pathology. B cells penetrating the CNS can enhance neuroinflammation through the production of autoantibodies or cytokines, whereas NK cells escalate inflammatory responses and neuronal damage via cytotoxic mechanisms (Lin et al. 2024).

### Immunometabolic Reprogramming of Microglia

Metabolic reprogramming is key to microglial activation and polarization. At homeostatic states, microglia favor oxidative phosphorylation as the mode of energy generation. Activated microglia, however, transition towards glycolysis, a metabolic reprogramming that facilitates the generation of ATP quickly and facilitates inflammatory response (Huang et al. 2024). This reprogramming is facilitated by enhanced glycolytic enzyme expression and downregulation of mitochondrial respiration (Huang et al. 2024). Lipid metabolism also has important effects on microglial function. Activated microglia contain lipid droplets, which reflect dysregulated cholesterol metabolism and defective lipid clearance. Such accumulation is associated with inflammatory amplification and dysfunction in AD (Kang and Rivest 2012; Dou et al. 2024). Additionally, dysregulation of cholesterol homeostasis, partly controlled by APOE, also promotes microglial inflammatory conditions and resultant AD pathology (Wu and Eisel 2023).

### Interaction between CNS-Resident and Peripheral Immune Cells

**Fig. 4:**
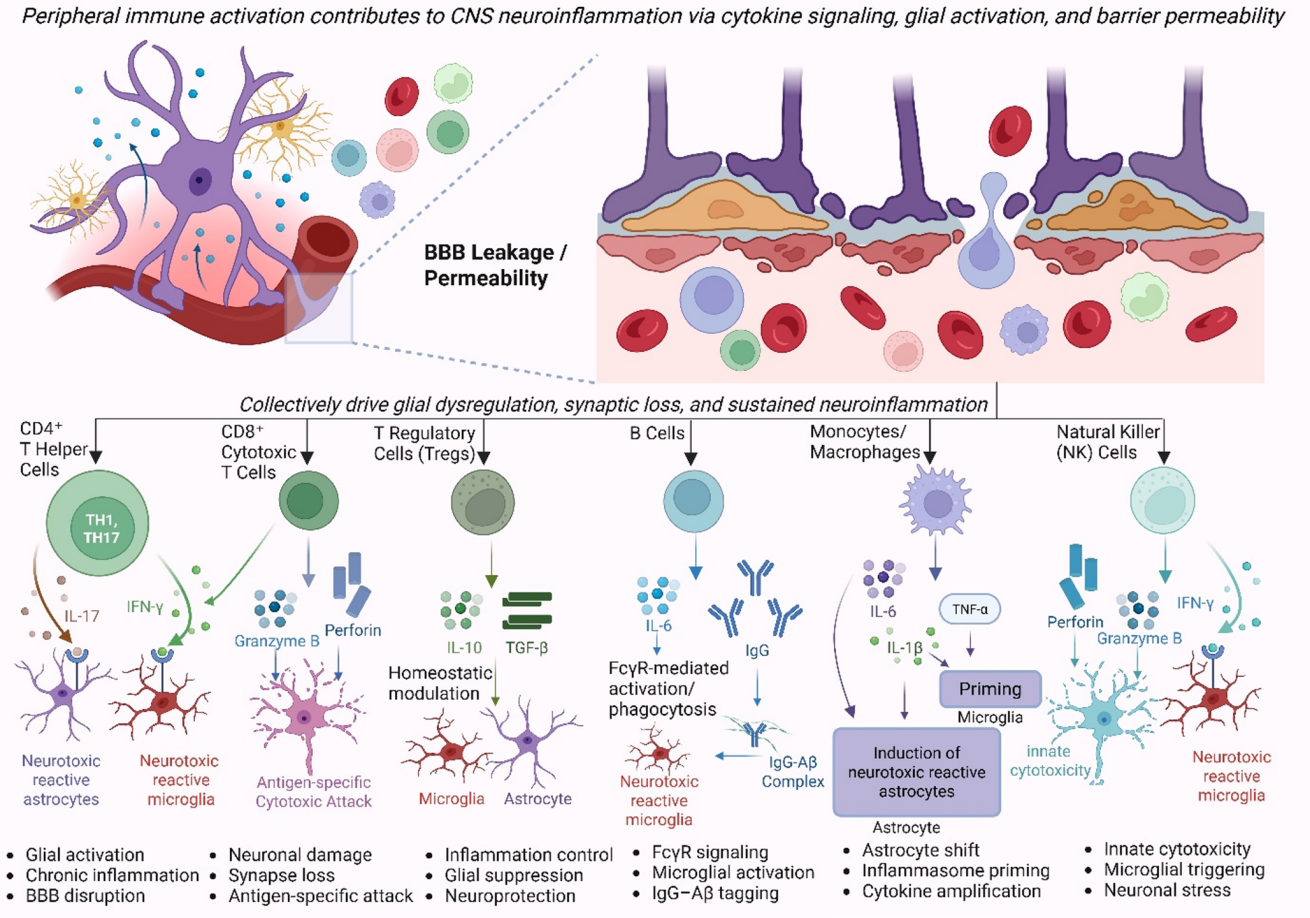
Peripheral–Central Neuroimmune Crosstalk in Alzheimer’s Disease. Peripheral immune activation contributes to central nervous system (CNS) neuroinflammation through a combination of cytokine signalling, glial activation, and disruption of the blood–brain barrier (BBB). The systemic immune cells, including CD4⁺ T helper cells, CD8⁺ cytotoxic T cells, regulatory T cells (Tregs), B cells, monocytes/macrophages, and natural killer (NK) cells, exert distinct effects on CNS-resident glial populations. CD4⁺ T cells release IL-17 and IFN-γ, which promote A1 astrocyte reactivity and M1 microglial activation. CD8⁺ T cells mediate antigen-specific cytotoxicity via perforin and granzyme B, leading to neuronal injury. Tregs suppress glial activation and inflammatory signaling through IL-10 and TGF-β. B cells produce IL-6 and secrete IgG antibodies that bind amyloid-beta (Aβ), leading to Fcγ receptor-mediated activation of microglia. Monocytes and macrophages infiltrate the CNS and release IL-1β, TNF-α, and IL-6, enhancing inflammasome priming and promoting A1 astrocyte conversion. NK cells contribute to microglial activation and neuronal stress through the release of IFN-γ, perforin, and granzyme B. BBB permeability facilitates immune cell infiltration and establishes sustained neuroimmune signaling, resulting in glial dysregulation, synaptic loss, and chronic neuroinflammation. Created in BioRender. Singh, D. (2025) https://BioRender.com/pqxd62m

Interactions between CNS-resident cells (like astrocytes, microglia) and invading peripheral immune cells establish intricate neuroimmune feedback loops. Activated microglia and astrocytes release cytokines like IL-1β, TNF-α, and IL-6, which also attract peripheral immune cells into the CNS, strengthening inflammation (Bhusal et al. 2022; Kim and Lee 2024). Invading peripheral immune cells additionally release other pro-inflammatory factors also, perpetuating a chronic neuroinflammation (Fig. 4). Cross-talk between the peripheral macrophages and microglia entails a signal exchange that impacts their activation status, as a result. Astrocytes also influence the peripheral immune cell activity through a secretion of chemokines (e.g., CCL2), which supports the leukocyte recruitment into our CNS (Fisher and Liddelow 2024). One must understand that continuous activation of these pathways leads to prolonged inflammatory states that play a critical role in the AD pathogenesis (Kodi et al. 2024).

## Therapeutic Implications and Drug Discovery

Therapeutic strategies for neuroimmune interactions in AD have received considerable attention based on the multifaceted role of microglial and astrocytic activation in disease progression. Neuroimmune therapies essentially attempt to modulate glial cell function, control inflammatory mediators, and restore metabolic balance (Müller et al. 2025). The regulation of inflammatory mechanisms, targeted metabolic therapy, and new therapeutic strategies employing cell-based and exosomal therapies hold great promise for effective Alzheimer’s disease treatments (Wu and Eisel 2023; Lin et al. 2024).

Cell-based approaches (allogeneic mesenchymal stromal cells). Early clinical studies in AD have primarily assessed feasibility and safety. For example, an open-label, intracerebroventricular hUCB-MSC (human umbilical cord blood mesenchymal stem cells) study in mild-to-moderate AD reported acceptable safety and exploratory biological effects but was not designed to demonstrate efficacy (Kim et al. 2021).

Randomized evidence (Phase 2a). In the CLEAR-MIND randomized, double-blind, placebo-controlled Phase 2a trial, allogeneic MSCs (laromestrocel; Lomecel-B) were tested in mild AD. The primary aim was safety; the study reported acceptable tolerability and exploratory signals on several secondary clinical measures (including a composite AD score, MoCA, and ADCS-ADL) at 39 weeks, while effect estimates and confidence intervals indicated that larger, confirmatory trials are required (Trial registration NCT05233774) (Rash et al. 2025). These data should be interpreted as early-phase and hypothesis-generating, not evidence of disease-modifying efficacy.

### Glial Activation and Inflammatory Mediators Targeting

Activation of microglia has a significant impact on AD pathology through inflammatory cascades. Therapies involve the modulation of microglial activation using inhibitors against pro-inflammatory signaling pathways, e.g., p38 MAPK inhibitors, and selective inhibition of receptors such as TREM2 (Li et al. 2023; Liu et al. 2019). The TREM2 agonists increase microglial phagocytosis and decrease inflammation, implying a therapeutic potential (Mohammad et al. 2024). Astrocytic protective mechanisms are also a potential therapeutic target. Glucagon-like peptide-1 (GLP-1) receptor agonists, such as liraglutide and semaglutide, have neuroprotective actions through the reduction of astrocytic inflammation and the promotion of neurogenesis and synaptic plasticity (Kopp et al. 2022). Similarly, the αB-crystallin, which is a molecular chaperone upregulated in reactive astrocytes, has shown neuroprotective actions through the inhibition of inflammatory pathways and maintenance of protein homeostasis (Bhusal et al. 2022). Nevertheless, NLRP3 inflammasome inhibitors including the MCC950, OLT1177 (dapansutrile), CY-09, and Tranilast, have been of interest due to their strong anti-inflammatory properties (shown in the Table 3). Summarily, MCC950 specifically inhibits NLRP3 formation (Coll et al. 2015), inhibiting IL-1β and IL-18 production, and has shown strong efficacy in preclinical models. OLT1177, which is an orally available NLRP3 inhibitor, is known to reduce the systemic inflammation and improve cognitive outcomes in various animal models. Studies show that CY-09 inhibits ATPase activity, which is essential for NLRP3 activation, whereas Tranilast is known to inhibit the inflammasome oligomerisation. So we can easily understand that these molecules offer us many high quality potential treatment options (Wu and Eisel 2023).

**Table 3:** Promising neuroimmune therapeutics in alzheimer’s disease

Therapeutic Agent	Target	Mechanism of Action	Clinical Status	References
MCC950	NLRP3 inflammasome	Selective inhibitor of NLRP3 that blocks ASC oligomerisation and IL-1β/IL-18 release	Preclinical (some halted early trials in humans)	(Coll et al. 2015; Dempsey et al. 2017; Naeem et al. 2024)
OLT1177 (Dapansutrile)	NLRP3 inflammasome	Selective small-molecule NLRP3 inhibition; suppression of IL-1β/IL-18 maturation	Preclinical efficacy in AD-model mice; clinical studies in non-AD indications	(Coll et al. 2015; Lonnemann et al. 2020))
CY-09	NLRP3 inflammasome	Binds NLRP3 NACHT domain, blocks ATP binding and inflammasome formation	Preclinical	(Jiang et al. 2017)
Tranilast	NLRP3 inflammasome	Prevents NLRP3 oligomerisation and downstream IL-1β secretion	Phase 2 (repurposed from fibrosis/allergy trials)	(Zhuang et al. 2020)
GLP-1 agonists (Liraglutide, Semaglutide)	GLP-1 receptor (CNS)	Enhance neurogenesis, reduce microglial and astrocytic activation	Phase 2 trials (e.g., NCT04123060 for AD)	(Zhao et al. 2022; Zheng et al. 2024)
αB-crystallin	Astrocytic & microglial inflammation	Chaperone protein that suppresses NF-κB and prevents pro-inflammatory cytokine release	Preclinical	(Adhikari et al. 2011)
Propranolol	β-adrenergic receptors	Reduces neuroinflammation via sympathetic tone suppression and microglial modulation	Preclinical	(Dobarro et al. 2013)
MSC-derived exosomes	Microglia, astrocytes	Deliver anti-inflammatory miRNAs (e.g., miR-22-3p, miR-223-3p); reduce glial activation and inflammasome signalling	Preclinical	(Guo et al. 2020; Zhai et al. 2021)
PD-1 checkpoint inhibitors	Peripheral T cells	Enhance peripheral immune surveillance; reduce neuroinflammation in transgenic AD models	Preclinical (tested in APP/PS1 mice)	(Manenti et al. 2022; Park et al. 2024)
hUCB-MSCs (intracerebroventricular)	Neuroinflammation (microglia/astrocytes)	Paracrine immunomodulation; trophic and pro-survival signaling; modulation of glial reactivity	Phase I open-label safety/feasibility in mild–moderate AD (ICV administration)	(Kim et al. 2021)
MSC-derived exosomes (BMSC-exos)	Microglial activation; synaptic support pathways (BDNF-related)	Extracellular-vesicle cargo (miRNAs/proteins/lipids) reducing neuroinflammation and enhancing neurotrophic signaling	Preclinical efficacy in AD-model mice (APP/PS1)	(Liu et al. 2022)
AL002 (TREM2 agonist monoclonal antibody)	TREM2 (microglia)	Agonism of TREM2–DAP12/SYK signaling to enhance microglial plaque engagement and disease-associated programs	Phase 1 first-in-human completed with target engagement; Phase 2 ongoing (NCT04592874)	(Long et al. 2024)
ANX005 (anti-C1q antibody)	C1q (classical complement pathway)	Neutralization of C1q to block initiation of classical complement and downstream C3/CR3-mediated synaptic pruning	First-in-human safety in healthy volunteers; investigational for neurodegeneration	(Lansita et al. 2017)

These promising therapeutic agents target the neuroimmune pathways in Alzheimer’s disease

### Metabolic Interventions

Targeting microglial immunometabolism offers us new and innovative therapeutic possibilities. Therapeutic modulation of microglial metabolic reprogramming from oxidative phosphorylation to glycolysis during activation can be mediated. This mediation is done by the AMPK and HIF-1α pathways (Baik et al. 2019). Activating AMPK, for instance, is known to enhance mitochondrial biogenesis, reduce dependency on glycolysis, and countering the inflammatory responses. Similarly, HIF-1α inhibitors can recapture glycolytic reprogramming, thereby reducing inflammatory cytokine synthesis and neuronal damage (Wu and Eisel 2023). Lipid metabolism pathways that are related to TREM2 and APOE show promising therapeutic targets. Research show augmentation of TREM2-regulated lipid metabolism has the ability to restore microglial lipid homeostasis (Wei et al. 2024). And thereby reducing lipid accumulation and inflammation. Treatments aimed at APOE, such as APOE modulators and lipid-lowering drugs, show promise in correcting cholesterol deregulation and decreasing the risk of Alzheimer’s disease, especially among APOE ε4 carriers (Williams et al. 2020). Pharmacologic repurification of metabolic drugs, such as GLP-1 receptor agonists (liraglutide, semaglutide) and beta-blockers (propranolol), is promising treatment patterns for Alzheimer’s disease. GLP-1 receptor agonists, originally approved in type 2 diabetes, promote neuronal well-being by enhancing anti-inflammatory activities, optimizing glucose metabolism, and promoting synaptic plasticity and neurogenesis (Femminella et al. 2019; Batista et al. 2018). Clinical trials indicate these agonists could slow cognitive impairment in Alzheimer’s patients by limiting microglial and astrocytic inflammation, adjusting mitochondrial activity, and enhancing neuronal insulin signaling (Femminella et al. 2019). Propranolol, classically employed for treating cardiovascular diseases, is beneficial in Alzheimer’s models by reducing stress-induced sympathetic nervous system activation, lowering peripheral and central inflammatory cytokines, and limiting oxidative neuronal injury, thereby maintaining cognitive functions (Miliotou et al. 2025). Repurposed drugs are valid adjunctive therapies that act on multiple pathogenic mechanisms of Alzheimer’s disease.

### Future Therapeutic Frontiers

Emerging treatment approaches for AD treatment, including the cell-based and exosomal therapies hold a great promise. Cell-based therapy using MSCs has been reported with encouraging results based on their inherent immunomodulatory and regenerative capabilities, and they were able to mitigate neuroinflammation and aid in neuronal repair (Duncan and Valenzuela 2017). MSC-derived EVs (like exosomes), is one of the cell-free therapeutic alternatives that can directly target therapeutic molecules such as microRNAs, proteins, and also the anti-inflammatory mediators into our CNS (Liu et al. 2022). These exosomes are capable of crossing the BBB and regulate microglial activation, astrocytic responses, and synaptic function. That is how they help in reducing AD pathology and cognitive impairment observed in preclinical models (Elia et al. 2019). Recent research work demonstrates the effectiveness of MSC-derived exosomes containing miR-146a and miR-124 in inhibiting neuroinflammatory pathways, enhancing synaptic plasticity, and improving cognitive performance in AD animal models (Elia et al. 2019). Additionally, targeting the peripheral immune system is becoming an attractive area of AD therapy. Peripheral immune modulation, like, the administration of inhibitors of programmed death-1 (PD-1) immune checkpoint, has been shown to control the infiltration and activity of immune cells into the CNS. Thus, decreasing neuroinflammation and cognitive dysfunction in model systems (Baruch et al. 2016). Also, regulation of peripheral immune cell migration and cytokine release, e.g., with the use of chemokine receptor antagonists and/or cytokine-neutralizing antibodies, would also further maximize therapeutic efficacy. This would be done by restricting harmful immune cell migration and inflammation within the CNS (Lin et al. 2024). Such approaches imply a widening therapeutic horizon for AD, coupling peripheral immune interventions with regenerative therapies for better clinical effectiveness.

## Leveraging AI and Machine Learning (AI/ML) in Neuroimmune Research

AI/ML in neuroimmunology has two complementary aims: (i) multimodal integration across single-cell, spatial, proteomic, lipidomic, and genetic layers to define disease-relevant glial states and targets, and (ii) clinical prediction that stratifies patients and forecasts progression for trial design. Exemplars of the first aim include an integrated, human AD multimodal cellular atlas linking cell states to pathology (Gabitto et al. 2024) and large-scale deep proteomic profiling showing disease modules not observed at RNA level (Johnson et al. 2022).

These resources provide biologically grounded features that AI models can use for endotyping and target nomination. Translational exemplars for the second aim demonstrate clinical feasibility in other domains. Delphi-2 M, a generative transformer trained on UK Biobank and externally validated in 1.9 M Danish records, forecasts rates for > 1,000 diseases with competitive performance; AIRE (AI-ECG) estimates individualized survival curves and incident cardiovascular risks from a single ECG across five international cohorts; Owkin’s multimodal survival model integrates imaging and clinical metadata to stratify prognosis in solid tumours; and TrajVis operationalizes AI trajectory models into decision support for chronic-disease management (Schutte et al. 2022; Li et al. 2024; Sau et al. 2024; Shmatko et al. 2025). These systems illustrate the real-world ingredients needed for deployment i.e., longitudinal data, external validation, calibration, and clinician-facing interfaces.

Analogous designs are now emerging in AD. Interpretable multimodal deep learning on ADNI (MRI + cognition + genetics) predicts MCI to AD conversion up to 4 years ahead with external testing (Wang et al. 2024), and new human brain resources (Gabitto et al. 2024)(SEA-AD) enable feature sets that tie glial states and lipid/proteome shifts to outcomes (Hawrylycz et al. 2024). Together, these support AI/ML frameworks that both discover targets from multi-omics and stratify patients for neuroimmune-focused trials.

**Table:** 

Box: Translational AI exemplars referenced in text	References
Delphi-2M (Nature, 2025): generative transformer trained on 0.4 M UK Biobank, validated on 1.9 M Danes; forecasts > 1,000 ICD-10 outcomes years ahead.	(Shmatko et al. 2025)
AIRE AI-ECG (Lancet Digital Health, 2024): single-ECG deep model with discrete-time survival; external validation across 5 cohorts.	(Sau et al. 2024)
Owkin multimodal survival (Eur J Cancer, 2022): imaging + clinical prognostic model with C-index ~ 0.71.	(Schutte et al. 2022)
TrajVis (JAMIA, 2024): clinician-facing visual decision support for AI trajectory models on longitudinal EHRs.	(Li et al. 2024)

### AI/ML Strategies for Disease Modelling and Drug Discovery

One of the core AI/ML approaches is predictive modeling (Bjerre et al. 2024). This approach helps in the identification of potential therapeutic targets in the intricate neuroimmune interactions. Also, the machine learning techniques like random forests, support vector machines (SVMs), and deep neural networks (DNNs) are especially suited for dealing with large-scale data (Ali et al. 2023). Therefore, this helps in uncovering the dormant or latent patterns in various neuroimmune pathways and predict possible drug targets. These predictive technologies use really large biological datasets from reported experimental and clinical research, providing a data-driven, strong method of discovering new interventional areas (Lin et al. 2024). Moreover, high-throughput screening (HTS) with the addition of AI/ML technologies greatly accelerates drug discovery (Wallach et al. 2024). Classical HTS techniques can be time-consuming and also labor-intensive. However, with the addition of AI/ML, researchers can now screen large chemical libraries against multiple neuroimmune targets in parallel. This is saving a lot of time and resources. AI-driven screening platforms leverage the upgraded algorithms to quickly evaluate molecular interactions, toxicity, efficacy, and likely off-target effects. This is enhancing the accuracy and speed of finding promising therapeutic leads significantly. For example, the recent AI-powered HTS investigations have effectively identified modulators that target microglial activation and inflammatory mediators in AD pathology (Maniam and Maniam 2024; Qiu and Cheng 2024; Mani et al. 2025). AI-augmented biomarker discovery represents a further area of importance in neuroimmune research. Biomarkers are critical for the early diagnosis, monitoring of disease, and assessment of therapeutic responses. Machine learning algorithms such as logistic regression, random forest classifiers, and convolutional neural networks (CNNs) offer advanced analytical tools to analyze complex clinical, imaging, genetic, and proteomic datasets (Chakraborty et al. 2024). These latest algorithms are capable of identifying subtle patterns and correlations that are mostly missed by our conventional analytical techniques. This progress in technology is presently leading to biomarkers with improved diagnostic accuracy, specificity, and high predictive value. For instance, ML-based analysis of fluid biomarkers and neuroimaging has successfully distinguished AD from other neurodegenerative diseases (Blanco et al. 2023; Cao et al. 2025). Moreover, with latest upgradations AI/ML has the capability of predicting disease progression rates accurately, and identified subgroups of patients that are likely to respond to particular therapies (Dixon et al. 2024; Jiang et al. 2025). Therefore, it is also considered as a big leap in precision medicine. Nevertheless, AI-assisted discovery of biomarkers enables personalization of therapy at various levels of treatment. Through stratification of patient populations according to specific biomarker profiles, clinicians are able to personalize treatments to aim for specific patient profiles to maximize therapeutic benefit while reducing side effects. This approach greatly facilitates the promotion of precision medicine in the treatment of AD as well as in neuroimmunology. Such use of AI and ML technology in neuroimmunology, apart from rebirthing the present, may also expedite scientific research as well as therapy development for various other disorders. These approaches further enhance our ability to comprehend complex interactions between the immune system and neuronal function and enable novel targets, efficacious therapeutics, and specific diagnostic biomarkers to be identified. Ongoing integration and innovation of AI/ML technologies into neuroimmune research have immense potential to revolutionize our ability to deal with and eventually control the effects of Alzheimer’s disease.

### Integrative Multi-omics Analysis

Single-cell RNA sequencing (or scRNA-seq) and spatial transcriptomics allow us now an unprecedented resolution in the profiling of neuroimmune cellular heterogeneity. The AI/ML algorithms help in easing the analysis of these high-dimensional data, enabling identification of distinct cell subtypes, gene regulatory networks, and disease-specific cell states. Additionally, methods such as t-distributed stochastic neighbor embedding (t-SNE) and Uniform Manifold Approximation and Projection (UMAP) are helping in visualizing and interpreting complex single-cell datasets (Lin et al. 2024; Roca et al. 2023). Immuno-metabolomics and the lipidomics, profile immune-related metabolic pathways and changes in lipid metabolism, respectively. These give essential insights into microglial and astrocytic functions. Combining these datasets with AI-assisted analysis allows one to have an integrated understanding of metabolic dysregulation in the neuroinflammation process. Machine learning algorithms like partial least squares discriminant analysis (or PLS-DA) and hierarchical clustering optimize the detection of metabolic biomarkers and therapeutic targets in different neuroimmune pathways (Wu and Eisel 2023; Worley and Powers 2016). Also, the AI/ML-based multi-omics data integration to detect new therapeutic pathways is related to applying AI/ML-based integration of multi-omics data. This includes genomics, transcriptomics, proteomics, and metabolomics and helps to identify interactive networks and regulation pathways underlying the neuroimmune interactions. Network-based analysis, Bayesian networks, and graph neural networks systematically recognize new biological pathways to offer possible innovative therapeutic targets in AD (Qiu and Cheng 2024).

### Future Directions in AI/ML Integration

**Fig. 5:**
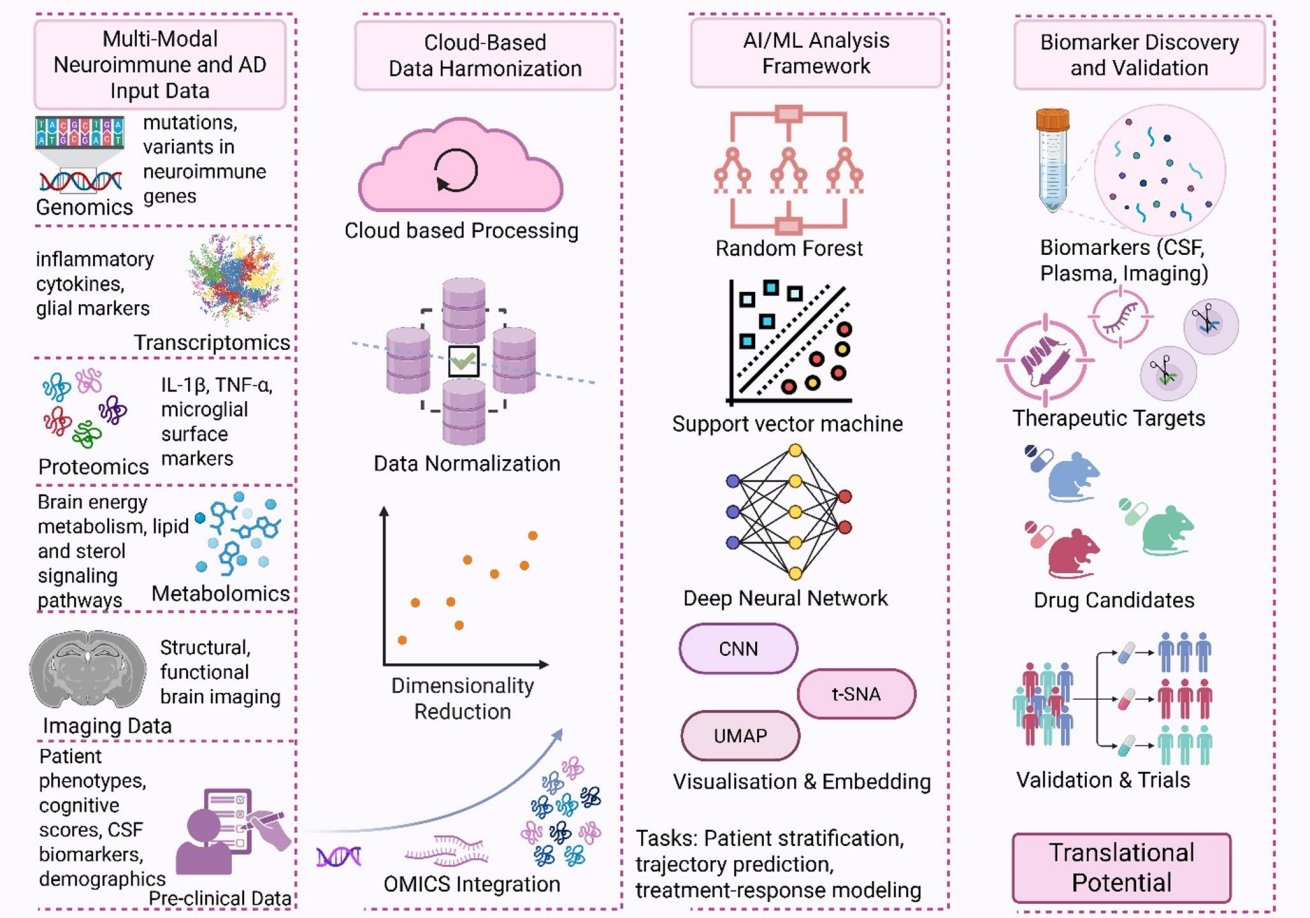
AI and Machine Learning-Enabled Pipeline for Neuroimmune Drug Discovery in Alzheimer’s Disease This diagram illustrates a translational pipeline for identifying neuroimmune biomarkers, therapeutic targets, and drug candidates in Alzheimer’s disease using artificial intelligence (AI) and machine learning (ML). Multi-modal input data including genomics, transcriptomics, proteomics, metabolomics, imaging, and clinical phenotypes are integrated through cloud-based data harmonisation workflows. These include data normalisation, dimensionality reduction, and omics integration. Machine learning algorithms such as random forest, support vector machines, deep neural networks, and visualisation tools including CNNs, t-SNE, and UMAP enable predictive modelling and feature extraction. These models generate testable hypotheses including candidate biomarkers, druggable targets, and compounds for therapeutic modulation. Outputs undergo experimental validation and clinical trial assessment, with translational potential extending to personalised treatment approaches in Alzheimer’s disease. Created in BioRender. Singh, D. (2025) https://BioRender.com/djukf6c

While both ML and AI offer substantial potential to develop neuroimmune studies in AD, some substantial hurdles need to be overcome for this to become a full reality. Top of these are heterogeneity of the data, scarce availability of good-quality, standard datasets, and challenges to interpreting advanced AI algorithms (Fig. 5). Variation in data collection protocols, heterogeneous analysis approaches, and varying standards make study integration and interpretations meaningful only across studies challenging. All these difficulties must be overcome through the implementation of standard data acquisition protocols, comprehensive repositories, and data quality and interoperability guidelines across research teams and platforms (Lin et al. 2024; Sedlakova et al. 2023). One of the critical challenges is also the interpretability and transparency of advanced AI models, typically so-called “black box” models. Deep learning architectures, by their intrinsic complexity and untransparent nature, can be discouraged from being applied in clinical environments and research arenas, where reasons behind predictions have to be understandable. The technology that furnishes interpretable and transparent outcomes from AI models, known as Explainable AI (XAI), offers one such solution (Shaughnessy et al. 2024). The XAI techniques like SHapley Additive exPlanations (SHAP) and Local Interpretable Model-Agnostic Explanations (LIME) can be used to explain the decision-making of the AI models (Hassan et al. 2025), making them easier to adopt, use and verify in neuroimmune research at multiple levels. These are the latest techniques which are in development and improvement mode and hold a lot of potential in neuroimmune research.

Nevertheless, new computational approaches like transfer learning and federated learning provide further enhancements in AI model generalizability and usability (Berkani et al. 2025). Transfer learning enables using pre-trained models to efficiently process smaller, disease-specific datasets prevalent in neuroimmune research. Federated learning, which supports collaborative analysis without violating data privacy, can support the creation of robust AI models by aggregating data from diverse sources. Moreover, cloud-based analytical platforms and automated data processing pipelines enable in smooth integration, analysis, and also the sharing of intricate multi-omics datasets. This helps in greatly promoting collaboration, reproducibility, and accessibility across the global research community (Lin et al. 2024).

## Challenges and Future Directions

The advancement of neuroimmune-targeted therapies for AD is confronted with major challenges owing to the intricacy of immune interactions in the CNS, interindividual heterogeneity, and nonetheless the dynamic nature of bioinformatics tools. While recent progress in glial biology, peripheral immune contributions, and AI-based research approaches has unveiled new therapeutic opportunities, there are some lingering challenges that require systematic resolution. In this review article, we have tried to address limitations and complexities in targeting neuroimmune mechanisms, highlight the significance of personalized medicine strategies, and recommend suggestions for future studies. Importantly, it is essential to address several limitations and complexities in targeting neuroimmune mechanisms. Perhaps one of the key challenges in targeting neuroimmune mechanisms is the dualistic role of glial activation in AD (Ebrahimi et al. 2025). Microglial and astrocytic responses are extremely context-dependent, in simpler words, initially protective but eventually deleterious (von Bernhardi and Eugenín 2025). Therapeutically manipulating these cells without impairing their physiological protective function is still challenging (Wu and Eisel 2023). Moreover, modulation of neuroimmune pathways is usually confronted with the challenge of redundancy and compensatory mechanisms in immune signaling. Inhibition of one inflammatory pathway can activate different pathways in an unintentional manner, which might exert unintended effects. Additionally, peripheral immune cell invasion and dynamic interactions with CNS-resident cells add complexity to the intervention strategies (Lin et al. 2024). The BBB continues to pose a major impediment to drug delivery to CNS. Although the BBB is usually breached in AD, its degree of dysfunction is heterogeneous, affecting therapeutic penetration and efficacy variably in different patients. Lastly, a significant limitation is the deficiency of robust animal models that perfectly capture the complexity of human neuroimmune interactions in AD. Variations in the biology of glial cells between rodents and humans restrict translational potential for preclinical results (Mohammad et al. 2024).

**Fig. 6:**
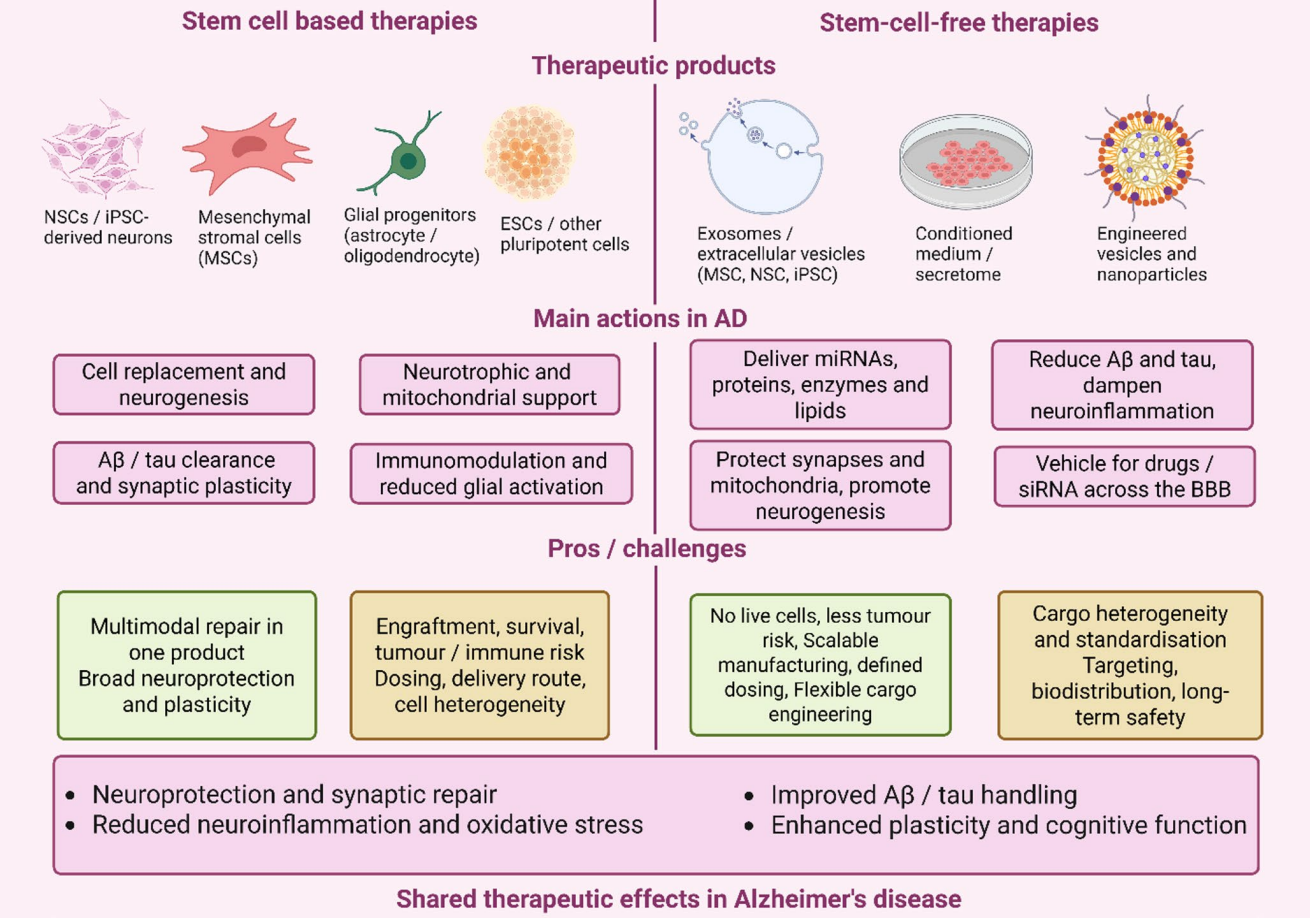
Comparison of stem cell based and stem-cell-free therapies for Alzheimer’s disease Schematic overview of therapeutic strategies derived from stem cell technology. Left, stem cell-based approaches use transplantation of NSCs or iPSC-derived neurons, mesenchymal stromal cells (MSCs), glial progenitors and other pluripotent cell sources. These cells can provide cell replacement and neurogenesis, support Aβ and tau clearance and synaptic plasticity, supply neurotrophic and mitochondrial support, and modulate neuroinflammation and glial activation. Right, stem-cell-free approaches exploit stem cell derived products, including extracellular vesicles and exosomes, conditioned medium or secretome, and engineered vesicles or nanoparticles. These therapies deliver miRNAs, proteins, enzymes and lipids, protect synapses and mitochondria, promote neurogenesis, reduce Aβ and tau pathology and dampen neuroinflammation, and can act as vehicles for drugs or siRNA across the blood–brain barrier. The lower panels summarise principal advantages and challenges of each strategy and highlight shared therapeutic effects in Alzheimer’s disease, including neuroprotection and synaptic repair, reduced neuroinflammation and oxidative stress, improved Aβ/tau handling and enhanced plasticity and cognitive function. Created in BioRender. Singh, D. (2025) https://BioRender.com/rcc79ot

Translating AI/ML into AD care requires (i) external validation across sites/cohorts, (ii) calibration for absolute risk, (iii) interpretability that is biologically grounded (linking to glial state features), and (iv) deployment tooling that meets clinician workflows (e.g., TrajVis-style interfaces). Recent exemplars include Delphi-2 M, AIRE, and Owkin that demonstrate these principles at scale and motivate similar multimodal, federated frameworks for ADNI and brain-bank datasets(Schutte et al. 2022; Sau et al. 2024; Shmatko et al. 2025). Personalized medicine is promising enormous potential in overcoming the heterogeneity witnessed in AD. The reason is due to its adaptability of therapeutic interventions to individual biological profiles. Biomarker-guided treatments are able to recognize particular neuroimmune dysregulation profiles in patients, making interventions more targeted. Such focused approach is known to remove many unwanted side effects. Novel biomarkers from CSF, blood samples, imaging methods, and multi-omics measurements (e.g., transcriptomics, proteomics) provide important information about patient-specific neuroimmune status. For example, single-cell RNA sequencing may define subtypes of disease-associated microglia (DAM), possibly guiding targeted immunomodulation (Lin et al. 2024). Notably, AI/ML algorithms can be added in such a way that it complements personalized approaches. This could be doing by predicting treatment outcomes, patient stratification based on molecular profiles, and appropriate clinical trial design. However, further validation of biomarkers in large heterogeneous cohorts is needed to determine robustness and clinical significance. To effectively close current areas of research neglect and propel AD advancement, several key future directions of research are proposed. Human-relevant experimental models must be generated; the employment of human-induced pluripotent stem cell (iPSC)-derived microglia and astrocytes, cerebral organoids, and advanced 3D co-culture systems would provide more specific and translationally relevant models to study complex neuroimmune interactions (Tiwari and Ginhoux 2025). They should be closely mimicing the human physiology and disease, thereby advancing our understanding of disease mechanisms and allowing us to determine effective therapeutic interventions. Figure 6 contrasts cell transplantation approaches with stem-cell-derived, cell-free therapies, and highlights their shared neuroprotective actions in Alzheimer’s disease.

Moreover, longitudinal multi-omics profiling encompassing genomics, transcriptomics, proteomics, metabolomics, and lipidomics, over the trajectory of AD can reveal dynamic and context-dependent changes in neuroimmune networks. Assembly of these dense data sets would also allow for more profound understanding of molecular and cellular alterations, enlightening us regarding the identification of key intervention points. State-of-the-art imaging methods, such as spatial transcriptomics and high-resolution imaging technologies, would also complement these strategies through imaging neuroimmune interactions in situ at cellular and molecular resolution (Jain and Eadon 2024). Incorporating AI/ML into these research frameworks will dramatically enhance the interpretability of various datasets, forecast disease trajectories, and identify novel therapeutic targets. Interpretable AI models capable of processing complex and heterogeneous biological streams of data are a powerful tool for unmasking hidden patterns and interactions among neuroimmune pathways. Stringent validation of potential biomarkers by multi-center clinical trials on heterogeneous patient populations is necessary for generalizability and clinical relevance. Furthermore, investigation of therapies that modulate peripheral immune functions and their effects on central nervous system inflammation represents a promising line of therapeutic development. Lastly, the construction of holistic combination therapies combining immunomodulation, metabolic interventions, neuroprotection, and clearance of pathological aggregates can greatly maximize treatment efficacy, thus moving toward personalized and more effective neuroimmune-targeted treatments for AD (Table 4).

**Table 4:** Machine learning applications in alzheimer’s disease research: methods, datasets, and translational insights

Application	Method/technique	Key datasets and resources	Advantages and limitations	References
Immune Cell Phenotyping	Deep learning models (e.g., CNNs, autoencoders)	Single-cell RNA-seq datasets, Human Cell Atlas	High-resolution phenotyping; limited by dataset heterogeneity and batch effects	(Rood et al. 2024)
Spatial Transcriptomics Analysis	Spatially resolved transcriptomics with Machine learning clustering	10x Genomics Visium, NanoString GeoMx DSP	Enables spatial mapping; limited resolution in certain platforms	(Ma et al. 2024; Wang et al. 2022)
Predictive Models for Inflammasome Inhibitors	Random Forest, Support Vector Machines (SVM)	LINCS, DrugBank	Accelerates drug discovery; potential for model overfitting	(Jiang et al. 2017)
Multi-Omics Data Integration	Graph neural networks, Bayesian networks	Alzheimer’s Disease Neuroimaging Initiative (ADNI), ROSMAP	Reveals complex regulatory networks; requires harmonization of datasets	(Kodam et al. 2023; Meng et al. 2024; Wang et al. 2021)
Clinical Trial Stratification	Logistic regression, survival analysis models	ADNI, European Medical Information Framework for AD (EMIF-AD)	Improves trial design; limited by initial cohort size and data variability	(Musto et al. 2023; Tam et al. 2022; Yi et al. 2023)

This table summarises key applications of artificial intelligence and machine learning across diverse stages of Alzheimer’s disease (AD) research, from single-cell immune phenotyping and spatial transcriptomic mapping to predictive drug modelling, integrative multi-omics analysis, and clinical trial stratification. Each application is linked to specific computational techniques and supported by relevant datasets such as the Human Cell Atlas, LINCS, DrugBank, ADNI, and ROSMAP. Strengths and limitations are outlined to reflect current capabilities and challenges, particularly in the context of biological heterogeneity, data harmonisation, and model generalisability. The selected references include primary research articles and domain-specific reviews that exemplify both foundational and emerging contributions to the field

## Conclusions

The recent advances in neuroimmune research have revolutionized our comprehension of AD also as an intricate interaction among CNS-resident cells (microglia and astrocytes) and peripheral immune processes. Understanding the dualistic functions of glial cells, the role of immune cell invasion, and the regulation of inflammatory signaling pathways has significantly elucidated the multifactorial aspect of AD pathogenesis. At the systems and molecular level, dysregulated neuroimmune communication not only worsens neuronal injury but also offers actionable targets for treatment. The relevance of integrative therapeutic strategies against neuroimmune dysregulation is becoming increasingly clear. Modulating microglial and astrocytic activation, remediating metabolic imbalances, and addressing peripheral immune crosstalk are multi-faceted strategies that synergistically can slow or arrest AD. NLRP3 inflammasome inhibitors, GLP-1 receptor agonists, and different strategies modulating immunometabolic pathways further exhibit encouraging preclinical and early clinical evidence. In addition, novel therapies with mesenchymal stem cell-derived exosomes and peripheral immune component immunomodulation hold promising promise for future translational efficacy. Predictive modeling with AI assistance, high-throughput screening, biomarker identification, and integration of multi-omics enable unraveling of incredibly complex biological networks. These platforms enable the identification of novel targets for therapy, optimal clinical trials, and attainment of personalized medicine strategies. New computational architectures, such as explainable AI and federated learning, can help to bridge existing limitations by enhancing model explainability and generalizability. In the future, a unified emphasis on longitudinal multi-omics research, validation of stable biomarkers, and the establishment of human-relevant experimental models will be critical. Speculatively, integration of multi-modal data sources (e.g., imaging, genomics, proteomics) into AI/ML platforms might enable earlier diagnosis, improved patient stratification, and more successful therapeutic intervention. Translation of these advances into concrete clinical benefits in the future will depend on upcoming interdisciplinary partnerships among neuroscientists, immunologists, bioinformaticians, and AI experts.

## Data Availability

Not applicable.

## Abbreviations

AD: Alzheimer’s disease
Aβ: Amyloid-β
NFT: Neurofibrillary tangle
CNS: Central nervous system
BBB: Blood–brain barrier
DAM: Disease-associated microglia
HAM: Human Alzheimer’s microglia
DAMP: Damage-associated molecular pattern
NF-κB: Nuclear factor-κB
NLRP3: NOD-, LRR- and pyrin domain-containing protein 3
ASC: Apoptosis-associated speck-like protein containing a CARD
CSF1R: Colony-stimulating factor 1 receptor
CR3: Complement receptor 3 (CD11b/CD18)
C1q: Complement component 1q
C3: Complement component 3
P2Y12: Purinergic receptor P2Y12
P2Y6: Purinergic receptor P2Y6
SYK: Spleen tyrosine kinase
TAM (RTKs): Tyro3/Axl/Mer receptor tyrosine kinases
Gas6: Growth-arrest–specific 6
ProS: Protein S
APOE: Apolipoprotein E
CLU (ApoJ): Clusterin (apolipoprotein J)
ROS: Reactive oxygen species
NO: Nitric oxide
IL-1β: Interleukin-1β
IL-6: Interleukin-6
IL-18: Interleukin-18
TNF-α: Tumour necrosis factor-α
IL-4: Interleukin-4
IL-10: Interleukin-10
TGF-β: Transforming growth factor-β
CSF: Cerebrospinal fluid
GFAP: Glial fibrillary acidic protein
NfL: Neurofilament light chain
sTREM2: Soluble TREM2
YKL-40: Chitinase-3-like protein 1
APP/PS1: Amyloid precursor protein/presenilin-1 transgenic mouse model
TLR: Toll-like receptor
RAGE: Receptor for advanced glycation end products
CD36: Scavenger receptor CD36
CD14: Co-receptor CD14
Itgax: Integrin alpha X (CD11c)
LPL: Lipoprotein lipase
Cst7: Cystatin F
CCL2: C-C motif chemokine ligand 2
IFN-γ: Interferon-γ
PD-1: Programmed death-1
EV: Extracellular vesicle
GLP-1: Glucagon-like peptide-1
AMPK: AMP-activated protein kinase
HIF-1α: Hypoxia-inducible factor-1α
PBMC: Peripheral blood mononuclear cell
GSDMD: Gasdermin-D
D4T: Stavudine
BACE1: β-site APP-cleaving enzyme 1
SP1: Specificity protein 1
Sox9: SRY-box 9
TXNIP: Thioredoxin-interacting protein
MoCA: Montreal Cognitive Assessment
ADCS-ADL: Alzheimer’s Disease Cooperative Study–Activities of Daily Living
ICV: Intracerebroventricular
AI: Artificial intelligence
ML: Machine learning
MRI: Magnetic resonance imaging
PET: Positron emission tomography
ADNI: Alzheimer’s Disease Neuroimaging Initiative
MCI: Mild cognitive impairment
CNN: Convolutional neural network
SVM: Support vector machine
t-SNE: t-Distributed stochastic neighbor embedding
UMAP: Uniform Manifold Approximation and Projection
PLS-DA: Partial least squares discriminant analysis
XAI: Explainable artificial intelligence
SHAP: SHapley Additive exPlanations
LIME: Local Interpretable Model-Agnostic Explanations
HER: Electronic health record
SEA-AD: Seattle Alzheimer’s Disease Brain Cell Atlas
LINCS: Library of Integrated Network-based Cellular Signatures
ROSMAP: Religious Orders Study and Memory and Aging Project
EMIF-AD: European Medical Information Framework for Alzheimer’s Disease
